# ASSOCIATION BETWEEN COUNTY TEMPERATURE AND LIMITATIONS IN ADLS IN A REPRESENTATIVE SAMPLE OF 1.7 MILLION OLDER ADULTS

**DOI:** 10.1093/geroni/igae098.2318

**Published:** 2024-12-31

**Authors:** Esme Fuller-Thomson, Holly Brooks, Elysia Fuller-Thomson, Andie MacNeil

**Affiliations:** University of Toronto, Toronto, Ontario, Canada; University of Toronto, Toronto, Ontario, Canada; University of Toronto, Toronto, Ontario, Canada; University of Toronto, Toronto, Ontario, Canada

## Abstract

Few studies have explored whether there is an association between area temperature and limitations in Activities of Daily Living (ADLs) such as serious difficulty with bathing and dressing. The objectives of this study were: (1) To examine the relationship between average county temperature during the coldest month of the year (i.e., January or February) (< 27°F, 27-31.9°F; 32-36.9°F; 37-41.9°F; ≥42°F) and the prevalence and adjusted odds of limitations in ADLs; and (2) To examine the relationship between the area’s hottest month’s average temperature (< 70°F, 70-74.9°F, 75-79.9°F, ≥80°F) and the prevalence and adjusted odds of limitations in ADLs. The sample included 1.7 million adults aged 65+ who responded to the nationally representative American Community Survey (2012-2017). After adjustments for age, sex, education, household income, race and time zone, there was a robust dose-response relationship indicating those living in areas with a higher average temperature during the coldest month had higher odds of limitations in ADLs. For example, in comparison to older adults living in regions where the coldest month’s average temperature was < 27°F, those living in areas where the temperature was slightly above freezing (32-36.9°F) had 22% higher adjusted odds of ADL limitations (OR=1.22; 95%CI=1.19, 1.25) and those living in regions where the temperature averaged ≥42°F had 33% higher odds (OR=1.33: 95%CI-1.30, 1.36). There was no significant relationship between the regional hottest month’s average temperature and ADL limitations. Future research is needed to explore why the absence of sub-freezing temperatures is associated with elevated odds of ADLs.

